# The Impact of Parental Personality on Birth Outcomes: A Prospective Cohort Study

**DOI:** 10.1371/journal.pone.0157080

**Published:** 2016-06-22

**Authors:** Naho Morisaki, Takeo Fujiwara, Reiko Horikawa

**Affiliations:** 1 Department of Social Medicine, National Research Institute for Child Health and Development, 2-10-1 Okura, Setagaya-ku, Tokyo, Japan; 2 Department of Global Health Promotion, Tokyo Medical and Dental University, 1-5-45 Yushima, Bunkyo-ku, Tokyo, Japan; 3 Department of Endocrinology, National Research Center for Child Health and Development, 2-10-1 Okura, Setagaya-ku, Tokyo, Japan; Hamamatsu University School of Medicine, JAPAN

## Abstract

**Objective:**

To investigate the effect of parental personality on birth outcomes.

**Design:**

Prospective cohort study.

**Setting:**

727 pregnant women and 579 spouses receiving antenatal care at a single-center in rural Tokyo, Japan during 2010–2013.

**Methods:**

We measured the association between maternal effect of parental personality traits assessed by the Cloninger’s Temperament and Character Inventory on birth outcomes, using multiple regression and adjusting for demographics.

**Results:**

Maternal self-transcendence personality was inversely associated with gestational age [-0.26 (95% confidence interval (CI): -0.51 to -0.01) weeks per unit] and positively associated with preterm birth [odds ratio (OR) 2.60 (95% CI: 1.00 to 6.75) per unit], while paternal self-transcendence personality was positively associated with gestational age [0.31 (95% CI: 0.07 to 0.55) weeks per unit]. Maternal reward dependence was positively associated with fetal growth [0.30 (95% CI: 0.02 to 0.59) per unit]. Other maternal and paternal personality traits associated with adverse maternal behavior, such as novelty seeking, harm avoidance and self-directedness, were not associated with birth outcomes.

**Conclusion:**

We found that specific parental personality traits can be associated with birth outcomes.

## Introduction

Preterm birth and restricted fetal growth have long been the most significant parameters of adverse outcomes in maternal-child health in many countries. Increasing knowledge has shown that their effects on children’s health are not limited to higher morbidity risk, [1] but long-term sequelae such as short stature [2], lower pulmonary function [2], higher blood pressure [2,3,4,5,6], lower insulin sensitivity [3], increased plasma low-density lipoprotein [5], and lower kidney function [7] leading to cardiovascular disease risk in later life [5,7,8].

Recent research has shown that various maternal behaviors such as smoking [9,10], inadequate eating habits [11,12], and physical and psychosocial stress from overwork or domestic violence [13,14,15,16], are known to be risk factors of preterm birth and low birth weight deliveries.

Previous studies measuring behavior using the Temperament and Character Inventory [17], a widely used psychobiological model that measures personality in seven dimensions (novelty seeking, harm avoidance, reward dependence, persistence, self-directedness, cooperativeness, and self-transcendence) have shown that personality is an underlying factor which determines behavior. For example, novelty-seeking personality was shown to be associated with smoking, and harm avoidance with nicotine dependence [18,19]. Depression has been reported to be associated with higher harm avoidance [20] and lower self-directedness [21] even among pregnant mothers [22,23], expectant fathers [23], and the general population [24].

However, no study using a valid personality assessment scale, including the two above, has assessed how birth outcomes are affected by maternal and paternal personality. Therefore, we examined the association between antenatal maternal and paternal personality and birth outcomes, such as preterm or small for gestational age (SGA), using the Temperament and Character Inventory [17].

## Material and Methods

### Study sample

This study protocol was approved by the Institutional Review Board at the National Center for Child Health and Development on August 2, 2010 (Study ID: H22-417). All participants provided written informed consent to participate in this study. Our study was done using a sub-cohort of the Seiiku Boshi Cohort, a prospective cohort study on pregnant women, their spouses and children. For this cohort, pregnant women attending antenatal visits at the National Center for Child Health and Development (NCCHD) from May 13, 2010 until Nov 28, 2013 were recruited during their first antenatal visit, which usually takes place in weeks 6–14 of gestation (N = 4,164). The NCCHD hospital is a tertiary hospital located in suburban Tokyo, and manages approximately 1,500 annual deliveries. Exclusion criteria were women whom nurses considered recruitment may be a burden, such as those diagnosed as threatened abortion. In total, 2,309 women consented to participate in this study. Participants were asked to fill out questionnaires, and medical records for the mother and child were retrieved from hospital charts for all deliveries at the hospital.

Among these women, a sub-group of women with single pregnancies were approached during their regular antenatal visits to additionally asked to fill out the Temperament and Character Inventory in the second trimester (N = 727). Of this sub-cohort, 579 spouses also completed the Temperament and Character Inventory.

### Measures

#### Measuring personality

We measured maternal and paternal personality in the second trimester using the Temperament and Character Inventory created by Cloninger [17,25], which was translated and validated in Japanese [26,27]. The inventory is a 4-point scaled 130-item questionnaire [26,27] designed to determine an individual’s range of personality traits across seven basic dimensions of temperament and character (each rated on individual scales of 1 to 4). We used the scale as a continuous variable.

Cloninger categorized personality into two domains of temperament and character [17]. ‘Temperament’ is described as automatic emotional responses to experience, which are thought to be biological and moderately stable throughout life. The following four dimensions describe temperament: novelty seeking, harm avoidance, reward dependence, and persistence. (20) ‘Character’ is described as one’s individual identity and differences in values and meaning that affect one’s perception of events, and is a domain that evolves and matures through experiences. Character also encompasses the psychology of personality development, and is defined by the following three measured dimensions: self-directedness, cooperativeness, and self-transcendence [17].

#### Demographic data

We collected maternal socio-demographic and other characteristic data and categorized them as follows: parental age, parity (0, 1 or more), annual household income (<4 million yen, 4–15 million yen, >15 million yen), maternal employment status (housewife, part-time job, full-time job), maternal education (bachelor degree or higher, some college credit, no college education), smoking during pregnancy (yes, no), and daily exercise during pregnancy (yes, no). We treated maternal and paternal age as continuous variables.

#### Birth outcomes

Gestational age as well as birth weight, height, and head circumference were obtained from medical charts. Fetal growth was calculated as gestational age-adjusted birth weight z-scores, using Japanese birth weight curves for reference [28].

#### Statistical analysis

First, we evaluated correlations among the seven personality traits for the participating women (i.e. four dimensions of temperament and three of character), as well as for the participating men. We also assessed cross-correlation between the personality traits of participating couples using Pearson’s correlation co-efficient.

Second, we used multivariate linear regression to assess whether each maternal/paternal personality dimension score was associated with birth outcomes, adjusting for parental socio-demographic characteristics. As maternal personality dimensions are inter-correlated, and maternal and paternal personality dimensions are also correlated, we repeated this analysis including all maternal characteristic traits in one model. We did not adjust for maternal complications, behaviors or mental disorders as we considered these caused by personality and would be mediators than confounders. All statistical analyses were conducted using the statistical software package Stata 13 (STATA Corp, College Station, TX), and the p-value <0.05 was considered as statistically significant when performing hypothesis tests.

## Results

Table 1 shows parental and infant characteristic distributions in our population. All mothers were married. The average maternal and paternal ages were 35.9 and 37.9 years, respectively. The majority (80%) of families had an annual household income of 4–15 million yen. Of the mothers, 50% had a job, 62% had a Bachelor’s degree, only 3.0% smoked, and 57% were nullipara. The preterm birth rate was 4.4%, the average infant birth weight was 3002g and 4.5% of infants were SGA.

**Table 1:** Parental and infant characteristics of 727 participating families.

	mean	SD
Parental characteristics		
Maternal age	35.9	4.1
Paternal age	37.9	5
	n	%
Annual household income		
<4 million yen	40	5.5
4–15 million yen	579	80
>15 million yen	86	12
Missing	22	3
Maternal employment status		
Housewife	341	47
Part time	119	16
Full time	246	34
Missing	21	2.9
Maternal education		
High school or lower	50	6.9
Some college	227	31
College or higher	448	62
Missing	2	0.3
Maternal smoking		
Yes	22	3
No	705	97
Parity		
0	415	57
1 or more	312	43
	mean	SD
Infant characteristics		
Birth weight (g)	3002	397.4
Head circumference (cm)	33.2	2.5
	n	%
Preterm delivery	32	4.4
Male sex	379	52
Small for gestational age	33	4.5

In Table 2 we show the correlation among maternal and Table 3 shows paternal personality traits, and cross-correlation between maternal and paternal personality traits were shown in Table 4. Correlation among the personality traits within one individual were very similar to findings previously reported by Cloninger [25] and others [29]. Among maternal personality traits, moderate correlation above 0.40 was observed between reward dependence and cooperativeness (0.54), as well as between self-directedness and cooperativeness (0.46), and negative moderate correlation below -0.40 was seen between self-directedness and harm avoidance (-0.48). Weaker but statistically significant correlations were observed among the majority of paired traits. The correlations among paternal personality traits were very similar to those of the maternal group, with the addition of harm avoidance and novelty seeking having a moderate negative correlation of -0.41.

**Table 2:** Correlation among maternal personality traits: Analysis of 727 mothers.

	NS	HA	RD	PS	SD	CO	ST
NS	1						
HA	-0.37*	1					
RD	-0.02	-0.05	1				
PS	-0.07	-0.18*	0.17*	1			
SD	-0.07*	-0.48*	0.23*	0.16*	1		
CO	-0.12*	-0.22*	0.54*	0.29*	0.46*	1	
ST	0.22*	-0.15*	0.07	0.22*	-0.05	0.10*	1

Correlations above 0.40 are indicated in bold.

* p<0.05

**Table 3:** Correlation among paternal personality traits: Analysis of 579 fathers.

	NS	HA	RD	PS	SD	CO	ST
NS	1						
HA	-0.41*	1					
RD	0.13*	-0.13*	1				
PS	0.00	-0.29*	0.05	1			
SD	0.04	-0.53*	0.16*	0.21*	1		
CO	0.03	-0.30*	0.45*	0.18*	0.41*	1	
ST	0.26*	-0.21*	0.06	0.31*	-0.05	0.14*	1

Correlations above 0.40 are indicated in bold.

* p<0.05

**Table 4:** Cross-correlation between maternal and paternal temperament: Analysis of 579 couples.

	NS	HA	RD	PS	SD	CO	ST
NS	0.05	-0.05	-0.01	0.04	0.03	0.06	0.05
HA	-0.05	0.15*	-0.02	-0.05	-0.13*	-0.08	-0.06
RD	-0.06	0.02	0.11*	0.01	0.04	0.11*	0.03
PS	0.04	-0.04	-0.01	-0.05	0.05	-0.04	-0.03
SD	-0.03	-0.08*	0.05	-0.01	0.12*	0.05	0.01
CO	-0.01	-0.03	0.10*	0.03	0.07	0.11*	0.05
ST	-0.01	-0.05	-0.05	0.11*	0.03	0.05	0.14*

Correlations above 0.40 are indicated in bold. NS, novelty seeking; HA, harm avoidance; PS, persistence; RD, reward dependence; SD, self-directedness; CO, cooperativeness; ST, self-transcendence

* p<0.05

Similarities could be seen among the personality traits of expectant mothers and fathers, with maternal and paternal harm avoidance, reward dependence, self-directedness, cooperativeness and self-transcendence having a significant weak correlation. Among the participating couples, one parent’s reward dependence was weakly but significantly correlated with the cooperativeness of the other, and the harm avoidance of one parent was weakly but significantly negatively correlated with the self-directedness of the other. In addition, maternal persistence and paternal self-transcendence were weakly negatively correlated.

In Tables 5 and 6, we show the estimated association between each parental personality trait and birth outcome. Maternal self-transcendence was significantly associated with lower gestational age [coefficient: -0.26 (95% confidence interval (CI): -0.51 to -0.01) weeks per unit] and preterm birth [odds ratio (OR): 2.60 (95% CI: 1.00 to 6.75) per unit]. This association persisted after adjustment for other maternal characteristics, including other personality traits. However, neither fetal growth (i.e. z score of birth weight adjusted for gestational age) nor risk of SGA was associated with maternal self-transcendence. Maternal reward dependence was associated with greater fetal growth [coefficient: 0.30 (95% CI: 0.02 to 0.59) per unit] but not SGA, after adjusting for other maternal personality traits and characteristics. Other maternal personality traits were not associated with birth outcomes.

**Table 5:** Impact of maternal personality traits on birth outcomes.

		Crude estimate				Adjusted for maternal and infant characteristics				Additionally adjusted for paternal temperament			
		effect	95% CI		p-value	effect	95% CI		p-value	effect	95% CI		p-value
Gestational length (weeks)	NS	0.00	-0.38	0.37	0.99	0.00	-0.36	0.37	0.99	0.04	-0.39	0.47	0.85
	HA	-0.07	-0.34	0.20	0.61	-0.10	-0.36	0.17	0.47	-0.14	-0.49	0.21	0.42
	RD	0.14	-0.20	0.48	0.43	0.04	-0.30	0.38	0.81	0.08	-0.32	0.48	0.70
	PS	-0.25	-0.49	-0.01	0.04*	-0.23	-0.46	0.01	0.06	-0.22	-0.47	0.04	0.09
	SD	0.23	-0.09	0.55	0.16	0.20	-0.11	0.52	0.21	0.11	-0.31	0.52	0.62
	CO	0.08	-0.33	0.49	0.70	0.03	-0.37	0.43	0.88	0.01	-0.53	0.54	0.98
	ST	-0.32	-0.56	-0.08	0.009**	-0.29	-0.52	-0.05	0.016*	-0.26	-0.51	-0.01	0.039*
Fetal growth (SD)	NS	-0.01	-0.27	0.24	0.92	0.01	-0.25	0.27	0.94	-0.03	-0.33	0.28	0.86
	HA	-0.09	-0.27	0.09	0.35	-0.05	-0.24	0.13	0.57	-0.05	-0.30	0.19	0.67
	RD	0.21	-0.02	0.45	0.08	0.24	0.00	0.48	0.047*	0.30	0.02	0.59	0.035*
	PS	0.09	-0.08	0.25	0.29	0.10	-0.07	0.26	0.26	0.08	-0.10	0.26	0.36
	SD	0.10	-0.12	0.32	0.37	0.05	-0.18	0.27	0.68	0.00	-0.29	0.30	0.98
	CO	0.09	-0.19	0.37	0.53	0.06	-0.23	0.34	0.70	-0.20	-0.57	0.18	0.31
	ST	0.05	-0.11	0.21	0.55	0.03	-0.14	0.19	0.74	0.00	-0.17	0.18	0.97
Preterm birth (odds ratio)	NS	0.79	0.23	2.78	0.71	0.50	0.12	2.00	0.33	0.39	0.07	2.16	0.28
	HA	1.02	0.42	2.49	0.97	1.10	0.42	2.95	0.84	1.21	0.33	4.40	0.77
	RD	0.43	0.14	1.36	0.15	0.45	0.13	1.61	0.22	0.34	0.80	1.42	0.14
	PS	2.53	1.13	5.65	0.02*	2.61	1.08	6.30	0.03*	2.33	0.87	6.24	0.09
	SD	0.92	0.31	2.71	0.88	1.13	0.36	3.50	0.84	1.53	0.32	7.33	0.59
	CO	0.81	0.20	3.20	0.76	0.98	0.22	4.34	0.98	0.83	0.11	6.28	0.86
	ST	2.60	1.18	5.74	0.02*	2.36	1.01	5.48	0.047*	2.60	1.001	6.75	0.049*
SGA (odds ratio)	NS	2.93	0.89	9.6	0.08	3.61	0.99	13.2	0.05	3.02	0.67	13.7	0.15
	HA	0.71	0.30	1.7	0.45	0.66	0.25	1.74	0.40	1.01	0.30	3.37	0.98
	RD	0.40	0.13	1.2	0.11	0.35	0.14	1.45	0.18	0.42	0.10	1.69	0.22
	PS	1.06	0.48	2.3	0.89	1.12	0.48	2.59	0.79	1.20	0.48	2.98	0.69
	SD	1.02	0.35	3.0	0.97	1.01	0.32	3.18	0.98	1.35	0.32	5.67	0.68
	CO	0.50	0.13	1.9	0.32	0.59	0.14	2.50	0.48	0.90	0.14	5.72	0.91
	ST	1.52	0.70	3.3	0.29	1.80	0.80	4.1	0.16	1.56	0.65	3.80	0.32

*p<0.05,

**p<0.01.

NS, novelty seeking; HA, harm avoidance; PS, persistence; RD, reward dependence; SD, self-directedness; CO, cooperativeness; ST, self-transcendence; SGA, small for gestational age, CI, confidence interval.

**Table 6:** Impact of paternal personality traits on birth outcomes.

		Crude estimate				Adjusted for maternal and infant characteristics				Additionally adjusted for paternal temperament			
		effect	95% CI		p-value	effect	95% CI		p-value	effect	95% CI		p-value
Gestational length (weeks)	NS	-0.17	-0.52	0.18	0.35	-0.22	-0.57	0.13	0.21	-0.18	-0.59	0.23	0.39
	HA	0.07	-0.18	0.31	0.59	0.12	-0.13	0.36	0.35	0.33	-0.01	0.66	0.06
	RD	-0.03	-0.35	0.29	0.86	-0.75	-0.39	0.24	0.64	-0.07	-0.43	0.28	0.68
	PS	0.19	-0.01	0.39	0.06	0.17	-0.03	0.36	0.10	0.09	-0.13	0.31	0.41
	SD	0.13	-0.17	0.42	0.39	0.11	-0.19	0.4	0.47	0.36	-0.02	0.73	0.06
	CO	0.19	-0.19	0.56	0.33	0.045	-0.33	0.42	0.82	-0.09	-0.55	0.37	0.71
	ST	0.22	0.00	0.44	0.047*	0.30	0.08	0.52	0.007*	0.38	0.13	0.62	0.002*
Fetal growth (SD)	NS	0.07	-0.20	0.34	0.61	0.05	-0.22	0.33	0.71	0.05	-0.27	0.37	0.77
	HA	-0.13	-0.31	0.06	0.19	-0.10	-0.29	0.09	0.30	-0.03	-0.30	0.24	0.81
	RD	0.11	-0.13	0.36	0.36	0.11	-0.13	0.34	0.36	0.02	-0.26	0.3	0.87
	PS	0.05	-0.10	0.20	0.52	0.06	-0.09	0.22	0.43	0.04	-0.13	0.22	0.63
	SD	0.18	-0.04	0.40	0.12	0.14	-0.09	0.37	0.23	0.03	-0.27	0.33	0.85
	CO	0.27	-0.02	0.55	0.06	0.26	-0.04	0.55	0.09	0.21	-0.15	0.58	0.25
	ST	-0.03	-0.19	0.14	0.76	-0.02	-0.18	0.15	0.85	-0.06	-0.25	0.13	0.52
Preterm birth (odds ratio)	NS	1.27	0.27	5.9	0.77	1.35	0.26	6.94	0.72	1.47	0.19	11.4	0.71
	HA	0.88	0.3	2.6	0.81	0.83	0.26	2.65	0.75	1.26	0.22	7.19	0.8
	RD	1.41	0.34	5.81	0.64	1.45	0.28	7.43	0.66	1.53	0.26	9.02	0.64
	PS	0.68	0.26	1.76	0.43	0.65	0.22	1.96	0.45	0.57	0.16	2.02	0.38
	SD	2.03	0.56	7.37	0.28	2.28	0.56	9.32	0.25	3.99	0.57	27.7	0.16
	CO	0.82	0.16	4.25	0.81	0.84	0.13	5.49	0.86	0.48	0.05	4.99	0.54
	ST	1.17	0.46	3.02	0.33	0.8	0.26	2.42.	0.69	1.08	0.3	3.94	0.9
SGA (odds ratio)	NS	1.28	0.34	4.81	0.72	1.46	0.37	5.72	0.59	1.22	0.23	6.38	0.82
	HA	0.87	0.34	2.21	0.77	0.85	0.31	2.36	0.76	0.89	0.20	3.86	0.88
	RD	0.99	0.3	3.3	0.99	0.98	0.28	3.48	0.98	1.28	0.30	5.51	0.74
	PS	0.80	0.36	1.78	0.58	0.74	0.32	1.71	0.48	0.7	0.27	1.82	0.46
	SD	1.11	0.36	3.37	0.86	1.19	0.37	3.85	0.77	1.63	0.34	7.87	0.54
	CO	0.65	0.16	2.68	0.55	0.59	0.13	2.72	0.50	0.41	0.06	3.01	0.38
	ST	1.08	0.47	2.46	0.86	1.01	0.4	2.51	0.99	1.14	0.39	3.29	0.81

*p<0.05.

NS, novelty seeking; HA, harm avoidance; PS, persistence; RD, reward dependence; SD, self-directedness; CO, cooperativeness; ST, self-transcendence; SGA, small for gestational age, CI, confidence interval.

Similarly, paternal self-transcendence was significantly associated with higher gestational age [0.31 (95% CI: 0.07 to 0.55) weeks per unit] but not risk of preterm birth or SGA, with the association persisting after adjustment for other maternal and paternal characteristics, including other personality traits. Other paternal personality traits were not associated with birth outcomes.

## Discussion

### Main findings

We investigated the association between maternal and paternal personality measured by the Temperament and Character Inventory, and birth outcomes. We found several traits of antenatal maternal and paternal personality to be a predictor of birth outcomes, especially gestational length. Higher maternal self-transcendence was associated with shorter gestational length and higher risk of preterm birth; higher maternal reward dependence was associated with greater fetal growth, and higher paternal self-transcendence was associated with longer gestational length. However, personality traits including novelty seeking, self-directedness, harm avoidance or cooperativeness associated with adverse behaviors (smoking) or mood disorders (depression) did not show significant association with neither fetal growth nor gestational length.

### Interpretation

Personality is an important factor for how people behave. As maternal and paternal behavior is likely to affect the fetus, we hypothesized that parental temperament would be associated with birth outcomes. However, the associations between temperament and birth outcomes that we observed could not be explained by known behaviors, such as smoking.

As for the association between smoking and maternal temperament, we found that novelty seeking, which is known to be associated with a decrease in basal dopamine and an increase in cluster B (impulsive) personality disorders [25], was associated with more smoking. In contrast, cooperativeness, which is known to be associated with less depression and less personality disorders [25], was associated with less smoking. However, although our study showed that although smoking was a predictor of both preterm birth [OR 3.6 (95% CI 1.2 to 11.6)] and SGA [OR 4.2 (95% CI 1.3 to 13.4)], neither novelty seeking or cooperation was related to preterm birth nor SGA. On the other hand, maternal self-transcendence and reward dependence, both of which were not related to smoking, were associated with preterm birth and fetal growth.

Ante-natal depression could also be a mediating factor between personality and birth outcomes, as higher harm avoidance [20,22] and lower self-directedness [22,29,30] have been reported to be associated with depression. However, it is still unknown whether maternal depression is associated with preterm birth or fetal growth, and we did not see any significant association between personality trait and birth outcomes. This discrepancy in findings suggests temperament may affect birth outcomes through pathways other than those mediated by known behavior. For example, the ability to adapt to change, follow externally cast norms, and the spouse’s capability to support her, all of which in part are determined by temperament, may be influencing how women cope with the stress of being pregnant, which in turn affects birth outcomes.

Self-transcendence, a character dimension of personality, is described as the experience of spiritual concepts; for example, regarding oneself as being at one with the universe [25]. People with high self-transcendence are described as spiritual and less materialistic with a tendency to have difficulties adapting in Western societies that value materialistic success [25]. This character dimension is also associated with bipolar disorder [31]. Therefore, women with higher self-transcendence who are less materialistic may be less likely to take supplement, such as multivitamin, which is reported to prevent preterm [32]. On the other hand, expectant fathers with higher self-transcendence may also have higher spirituality, which works as good mental supporters for their partners, leading to increased gestational length.

Reward dependence, an inheritable temperament dimension of personality, relates to social sensitivity and dependence on the approval of others [25]. People who rate highly in reward dependence are sensitive to social cues that facilitate affectionate social relations [25]. This dimension of temperament has been associated with lower basal noradrenaline [33] and decreased risk of cluster A personality disorders [34]. Therefore, women with higher reward dependence could be more anxious to follow the social norms of being a “good mother” during pregnancy, which may lead to greater fetal growth.

### Strengths and limitations

Our study has several limitations. First, as the study was conducted in an area of Japan with a fairly well-educated, relatively higher socioeconomic-status population, our findings may not be generalizable to other cultural backgrounds. However, some of our results did show similarities with previous studies, such as the correlation among personality traits of one individual [25,29]. Second, our sample size was limited and may not have enough power to detect all associations. For example, higher novelty seeking, which is related to smoking, showed an insignificant but large effect estimate on risk of SGA [OR 3.02 (0.67, 13.7) per unit]. Third, as we conducted analysis on each of the seven subscales of temperament, some of our findings showing statistically significant could be due to chance. However, the association between maternal self-transcendence and pregnancy duration was significant for both gestational length and risk of preterm birth, and the association between paternal self-transcendence and pregnancy duration was highly significant, suggesting a true relationship. However, the lack of previous studies has inherently limited the verification of our findings. Hence, larger studies are warranted.

## Conclusion

We found specific parental personality traits can be associated with birth outcomes, however failed to explain these observed relationships by behaviors associated with personality. Further research to elucidate how parental personality influence birth outcomes by altering behavior is needed.
